# Non-thermal plasma treatment improves chicken sperm motility via the regulation of demethylation levels

**DOI:** 10.1038/s41598-018-26049-5

**Published:** 2018-05-15

**Authors:** Jiao Jiao Zhang, Huynh Luong Do, Nisansala Chandimali, Sang Baek Lee, Young Sun Mok, Nameun Kim, Seong Bong Kim, Taeho Kwon, Dong Kee Jeong

**Affiliations:** 1 Laboratory of Animal Genetic Engineering and Stem Cell Biology, Department of Advanced Convergence Technology and Science, Jeju National University, Jeju, 63243 Republic of Korea; 2 Department of Chemical and Biological Engineering, Jeju National University, Jeju, 63243 Republic of Korea; 3 Plasma Technology Research Center, National Fusion Research Institute, Gunsan-si, Jeollabuk-Do 54004 Republic of Korea; 4 Laboratory of Animal Genetic Engineering and Stem Cell Biology, Subtropical/Tropical Organism Gene Bank, Jeju National University, Jeju, 63243 Republic of Korea

## Abstract

The quality of avian semen is an important economic trait in poultry production. The present study examines the *in vitro* effects of non-thermal dielectric barrier discharge plasma on chicken sperm to determine the plasma conditions that can produce the optimum sperm quality. Exposure to 11.7 kV of plasma for 20 s is found to produce maximum sperm motility by controlling the homeostasis of reactive oxygen species and boosting the release of adenosine triphosphate and respiratory enzyme activity in the mitochondria. However, prolonged exposure or further increase in plasma potential impairs the sperm quality in a time- and dose-dependent manner. Optimal plasma treatment of sperm results in upregulated mRNA and protein expression of antioxidant defense-related and energetic metabolism-related genes by increasing their demethylation levels. However, 27.6 kV of plasma exerts significant adverse effects. Thus, our findings indicate that appropriate plasma exposure conditions improve chicken sperm motility by regulating demethylation levels of genes involved in antioxidant defense and energetic metabolism.

## Introduction

Sperm quality of male chickens directly affects fertility and hatchability^1^, which are the most important economical requisites of poultry breeding because they determine the profitability of production. Chicken spermatozoa acquire the motility as soon as they leave the testis. Sperm requires high levels of adenosine triphosphate (ATP) as the energy source to ensure motility, which shows the ability of sperm to move properly and efficiently through the female’s reproductive tract to reach the egg^2^. The middle region of sperm contains numerous mitochondria to supply energy for flagellum movement. These biological features indicate a central role of energetic metabolism in sperm motility to ensure the successful fertilization and reproduction of male chickens.

Non-thermal dielectric barrier discharge (DBD) plasma is generated at atmospheric pressure in gas when a high voltage of time-varying waveform or short duration pulses is applied between two electrodes, which produces electrically safe plasma without substantial gas heating^3–5^. Advancements in non-thermal DBD plasma systems have promoted the potential for various medical and biological applications including sterilization, blood coagulation, wound healing, tissue regeneration, dental treatment, promotion of cell proliferation and transfection, and cancer treatment^5–9^. Our previous studies apply the non-thermal DBD plasma in the promotion of soybean sprout growth^10^ and the embryonic development of chicken during the early stage of incubation^11^. However, studies on the effects of non-thermal DBD plasma on sperm quality remain to be conducted. Thus, the present study addresses whether plasma treatment can affect chicken sperm parameters.

When cells or tissue surfaces are exposed to non-thermal DBD plasma a variety of biologically active reactive species are generated, particularly, reactive oxygen species (ROS)^4,12,13^. DBD plasma selectively initiates and amplifies ROS signaling to enhance cell proliferation and differentiation^14^; however, excessive intracellular ROS formation after DBD plasma treatment leads to cell deficiency^15^. ROS homeostasis plays an important role in sperm physiological processes, whereas elevated concentrations of ROS result in sperm morphological pathology, ATP depletion, lipid peroxidation, and loss of motility and viability^16^. Thus, the balance between ROS generation and scavenging activity is required to ensure optimum sperm quality. Here, we hypothesize that non-thermal DBD plasma may affect chicken sperm quality by mediating the ROS balance and energetic metabolism.

Sperm DNA methylation is a key regulator of transcription and contributes to altered gene expression that deteriorates semen properties, sperm counts, and motility^17,18^. Genes that promote development are generally hypomethylated in the sperm^19^, whereas hypermethylation can lead to impaired spermatogenesis and poor sperm quality^20^. Changes in sperm methylation patterns in response to toxic exposure can have severe consequences on chromatin integrity and gene expression profiles^21,22^. The pronounced hypermethylation observed in the radiation-exposed spermatozoa implies defective chromatin condensation, which in turn produces morphologically abnormal spermatozoa^23^. Hence, these findings prompt that changes in sperm quality are potentially influenced by changes in DNA methylation levels following plasma exposure.

Therefore, the effects of non-thermal DBD plasma on chicken sperm quality and the mechanisms that regulate the antioxidant defense and energetic metabolism system of sperm through cytosine methylation remain to be elucidated.

## Results

### Sperm quality

Our results show that sperm motility is improved within 40 s of non-thermal plasma exposure to 11.7 kV, peaking at 20 s (Table 1). There are no significant differences in sperm viability, acrosome integrity, DNA integrity, and total fertility between the control group and the plasma-treated groups within 40 s. However, plasma exposure for 60 s or longer has significant impairments on the sperm quality. These indicate a time-dependent effect of plasma treatment on sperm quality. Moreover, plasma is found to influence sperm motility in a dose-dependent manner. At 20 s of exposure, maximum sperm motility is obtained at 11.7 kV, with an increase of 0.23-fold (*p* < 0.001) compared with that of the control group (Table 2). However, sperm motility starts to decrease at plasma potentials higher than 16.4 kV. In addition, spermatozoa exposed to plasma at higher than 16.4 kV for 20 s exert inhibitory effects on viability, integrity of acrosome and DNA, and total fertility. However, exposure to 11.7 kV of plasma shows no significant changes in the abovementioned sperm properties (Table 2).

**Table 1:** Effect of plasma exposure for different durations on chicken sperm quality.

Exposure duration (s)	Sperm count ×10^9^/ml	Sperm viability (%)	Sperm motility (%)	Acrosome integrity (%)	DNA integrity (%)	Total fertility (%)
0	2.77 ± 0.05	85.21 ± 2.09	36.84 ± 1.35	35.35 ± 0.20	98.00 ± 0.41	84.00 ± 3.27
10	2.79 ± 0.01	85.06 ± 1.01	42.17 ± 1.85^**^	35.13 ± 2.63	97.17 ± 0.62	88.00 ± 3.27
20	2.78 ± 0.07	85.11 ± 0.56	45.42 ± 2.23^**^	35.23 ± 1.27	97.67 ± 0.24	89.33 ± 4.99
40	2.76 ± 0.08	84.16 ± 2.54	42.36 ± 1.75^**^	34.14 ± 1.93	97.00 ± 0.41	85.33 ± 1.89
60	2.77 ± 0.03	76.26 ± 2.89^**^	30.67 ± 1.19^**^	30.95 ± 1.86^*^	82.00 ± 0.82^**^	70.67 ± 4.99^**^
80	2.77 ± 0.06	66.23 ± 1.43^**^	25.58 ± 0.97^**^	27.44 ± 0.94^**^	66.33 ± 0.85^**^	54.67 ± 3.77^**^

Plasma exposure intensity was 11.7 kV. Values are expressed as the mean ± standard error (n = 10) of three replicates; n represents an individual cock. Within a column: ^*^p < 0.05 versus control; ^**^p < 0.01 versus control, according to one-way ANOVA and LSD test.

**Table 2:** Effect of plasma exposure at different potentials on chicken sperm quality.

Exposure potential kV)	Sperm count ×10^9^/ml	Sperm viability (%)	Sperm motility (%)	Acrosome integrity (%)	DNA integrity (%)	Total fertility (%)
0	2.77 ± 0.05	85.21 ± 2.09	36.84 ± 1.35	35.35 ± 0.20	98.00 ± 0.41	84.00 ± 3.27
11.7	2.78 ± 0.07	85.11 ± 0.56	45.42 ± 2.23^**^	35.23 ± 1.27	97.67 ± 0.24	89.33 ± 4.99
16.4	2.78 ± 0.02	77.33 ± 3.44^**^	35.92 ± 1.06	32.47 ± 1.30	87.67 ± 0.85^**^	78.67 ± 3.77
22.0	2.77 ± 0.07	71.65 ± 2.81^**^	31.55 ± 1.72^**^	32.05 ± 2.23	52.83 ± 1.84^**^	40.00 ± 3.27^**^
27.6	2.77 ± 0.02	65.84 ± 2.19^**^	24.38 ± 1.20^**^	30.43 ± 2.63^*^	38.83 ± 1.03^**^	14.67 ± 1.89^**^

Plasma exposure time was 20 s. Values are expressed as the mean ± standard error (n = 10) of three replicates; n represents an individual cock. Within a column: ^*^p < 0.05 versus control; ^**^p < 0.01 versus control, according to one-way ANOVA and LSD test.

**Figure 1:**
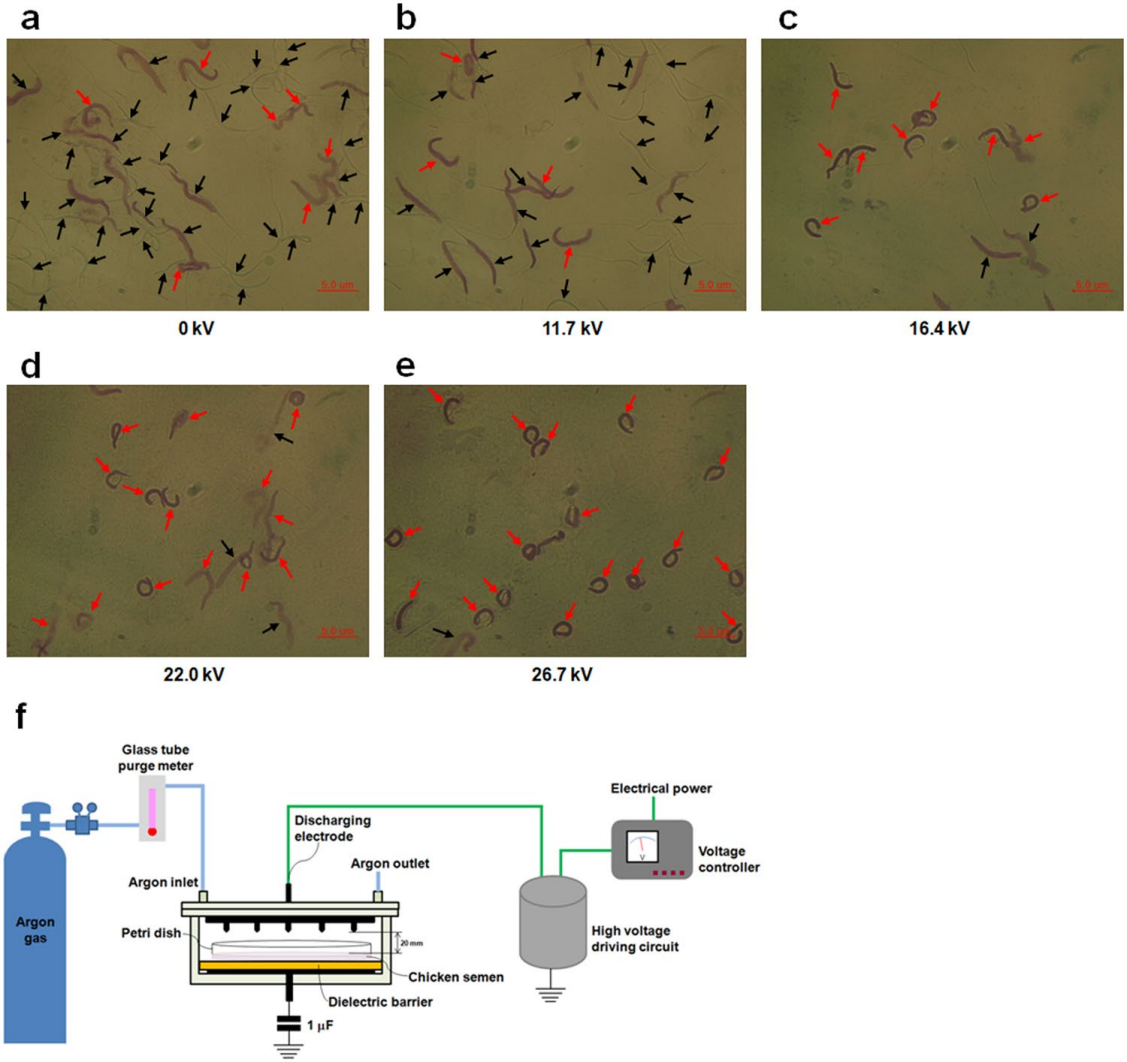
Effect of plasma on chicken sperm morphology. Semen of 60-week-old cocks was exposed to increasing potentials of plasma for 20 s. (**a**) 0 kV, (**b**) 11.7 kV, (**c**) 16.4 kV, (**d**) 22.0 kV, and (**e**) 26.7 kV. Semen smears were stained with Wright-Giemsa. Representative sperm morphology was photographed using a Leica DM 2500 microscope. Black arrows show normal spermatozoa and red arrows show abnormal spermatozoa. Scale bar: 5.0 μm. (**f**) Schematic of non-thermal DBD plasma treatment system.

The optical microstructure of spermatozoa exposed to different plasma potentials for 20 s is determined. Spermatozoa from the plasma-treated group at 11.7 kV and the control group show no significant differences in the ratio of abnormal spermatozoa, 84.44% of spermatozoa in the control group and 85.71% in the 11.7 kV group are normal and complete (Fig. 1a,b). However, exposure to potential higher than 16.4 kV exerts harmful effects on sperm morphology and produces a significant number of crooked spermatozoa (Fig. 1c–e). Plasma exposure at 27.6 kV shows the highest ratio of abnormal spermatozoa (94.44%; Fig. 1e), which increases by 5.07-fold compared with that of the control group (15.56%).

### ROS and antioxidant enzyme

ROS and malondialdehyde (MDA) levels in chicken spermatozoa decrease within 40 s of plasma exposure at 11.7 kV (see Supplementary Table S1). Superoxide dismutase (SOD), catalase (CAT), and glutathione peroxidase (GPx) activities increase within 40 s of plasma treatment. However, prolonged plasma exposure shows contrary effects on those parameters. Plasma treatment shows a dose-dependent effect on ROS and MDA levels and antioxidant enzyme activity in chicken spermatozoa (Table 3). At 20 s of exposure, minimal levels of ROS and MDA and maximum activities of antioxidant enzyme are observed at 11.7 kV, with decreases of 0.10- (*p = *0.019) and 0.33-fold (*p = *0.004) in ROS and MDA, respectively, and increases of 0.71- (*p* < 0.001), 0.60- (*p* < 0.001), and 0.42-fold (*p* < 0.001) in SOD, CAT, and GPx compared with those of the control group. On the other hand, further increases in plasma potential (16.4 to 27.6 kV) result in continuously elevated ROS and MDA levels but decreased SOD, CAT, and GPx levels (Table 3).

**Table 3:** Effect of plasma exposure at different potentials on ROS and antioxidant enzyme in chicken spermatozoa.

Exposure potential (kV)	ROS (nmole DCF/10^9^spz)	MDA (nmole/10^9^spz)	SOD (U/10^9^spz)	CAT (U/10^9^spz)	GPx (mU/10^9^spz)
0	6.47 ± 0.18	3.04 ± 0.18	42.53 ± 0.91	2.16 ± 0.11	4.34 ± 0.10
11.7	5.79 ± 0.18^*^	2.04 ± 0.17^**^	72.64 ± 3.17^**^	3.45 ± 0.23^**^	6.15 ± 0.31^**^
16.4	6.96 ± 0.22	3.12 ± 0.16	41.15 ± 1.79	1.99 ± 0.07	4.27 ± 0.19
22.0	10.89 ± 0.13^**^	3.65 ± 0.33^*^	36.48 ± 2.31^*^	1.74 ± 0.15^*^	4.04 ± 0.08
27.6	14.37 ± 1.52^**^	4.16 ± 0.09^**^	30.27 ± 1.55^**^	1.54 ± 0.16^**^	3.10 ± 0.24^**^

Plasma exposure time was 20 s. Values are expressed as the mean ± standard error (n = 10) of three replicates; n represents an individual cock. Within a column: ^*^p < 0.05 versus control; ^**^p < 0.01 versus control, according to one-way ANOVA and LSD test.

### ATP and mitochondria respiratory enzyme

ATP and nicotinamide adenine dinucleotide hydrogen (NADH) levels, and enzymatic activities of cytochrome c oxidase and ATP synthase increase within 40 s of plasma exposure at 11.7 kV, but longer plasma exposure duration decreases ATP production and respiratory enzyme activity (see Supplementary Table S2). A dose-dependent effect on ATP, NADH, and mitochondria respiratory enzyme in the spermatozoa is observed in the plasma-treated group. Maximum ATP, NADH, and respiratory enzyme levels are observed at 20 s of exposure at 11.7 kV, with increases of 0.61- (*p* < 0.001), 0.38- (*p* < 0.001), 0.63- (*p* < 0.001), and 0.45-fold (*p* < 0.001). Further increases in plasma potential decrease the ATP, NADH, and respiratory enzyme levels (Table 4).

**Table 4:** Effect of plasma exposure at different potentials on ATP and activity of mitochondria respiratory enzyme in chicken spermatozoa.

Exposure potential (kV)	ATP (nmole/10^9^spz)	NADH (U/10^9^spz)	Cytochrome c oxidase (mU/10^9^spz)	ATP synthase (mU/10^9^spz)
0	3.62 ± 0.15	0.57 ± 0.02	34.55 ± 0.93	21.53 ± 0.93
11.7	5.84 ± 0.40^**^	0.79 ± 0.02^**^	56.40 ± 3.16^**^	31.30 ± 2.38^**^
16.4	3.54 ± 0.24	0.56 ± 0.05	32.50 ± 0.97	19.45 ± 0.63
22.0	3.16 ± 0.31	0.42 ± 0.02^**^	24.36 ± 1.78^**^	14.29 ± 0.84^**^
27.6	2.62 ± 0.17^**^	0.32 ± 0.03^**^	14.43 ± 1.52^**^	10.24 ± 0.41^**^

Plasma exposure time was 20 s. Values are expressed as the mean ± standard error (n = 10) of three replicates; n represents an individual cock. Within a column: ^*^p < 0.05 versus control; ^**^p < 0.01 versus control, according to one-way ANOVA and LSD test.

### DNA methylation

**Figure 2:**
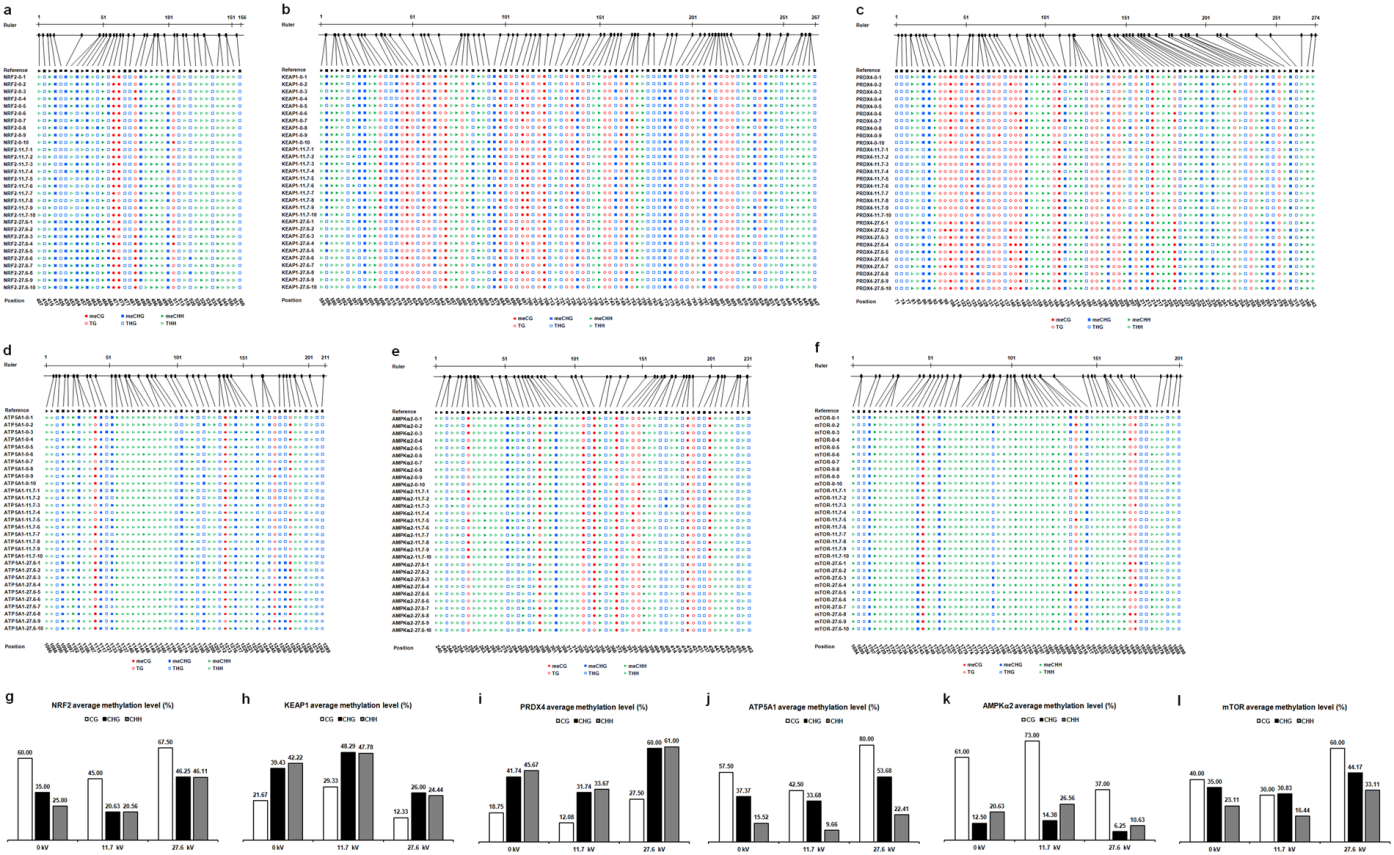
Cytosine methylation analysis of chicken spermatozoa. Cytosine methylation of (**a**) *NRF*2, (**b**) *KEAP1*, (**c**) *PRDX4*, (**d**) *ATP5A1*, (**e**) *AMPKα2*, and (**f**) *mTOR* were analyzed using CyMATE. Lengths of sequenced regions and position of cytosine are shown schematically. The order of the individual sequences of ten clones is listed on the left. 0 represents the control group; 11.7 represents plasma-treated group at 11.7 kV for 20 s; and 27.6 represents plasma-treated group at 27.6 kV for 20 s. The reference sequence is shown in the first line. The sequence is distinguished by circles for CG, squares for CHG, and triangles for CHH. Filled symbols represent methylated cytosine, and open symbols represent unmethylated cytosine. Average methylation levels for CG, CHG, and CHH of (**g**) *NRF2*, (**h**) *KEAP1*, (**i**) *PRDX4*, (**j**) *ATP5A1*, (**k**) *AMPKα2*, and (**l**) *mTOR*.

Bisulfite sequencing of *NRF*2, *KEAP1*, *PRDX4*, *ATP5A1*, *AMPKα2*, and *mTOR* is performed to determine the exact sites, type, and extent of methylation. Sequence analysis results of the target genes in the spermatozoa in the control and plasma treatment groups at 11.7 kV and 27.6 kV for 20 s using CyMATE software are shown (Fig. 2a–f). Methylation levels in the sequenced regions of the target genes show contradictory effects between samples treated with 11.7 kV and 27.6 kV of plasma. Exposure to 11.7 kV decreases methylation levels of *NRF2* (88/380, 23.16% methylation ratio), *PRDX4* (203/770, 26.36%), *ATP5A1* (109/520, 20.96%), and *mTOR* (123/610, 20.16%) 0.10-, 0.10-, 0.06-, 0.06-fold. On the other hand, methylation levels of *KEAP1* (386/920, 41.96%) and *AMPKα*2 (181/580, 31.21%) increase 0.08- and 0.06-fold, respectively (Table 5). However, methylation levels of *NRF2*, *PRDX4*, *ATP5A1*, and *mTOR* in the group treated with 27.6 kV of plasma show corresponding increases of 0.16-, 0.14-, 0.12-, 0.10-fold, whereas *KEAP1* and *AMPKα*2 methylation levels decrease 0.13- and 0.11-fold, respectively (Table 5). In addition, the variations in average methylation levels of *ATP5A1*, *mTOR*, and *AMPKα*2 for CG type are found to be more pronounced than those of CHG and CHH in the spermatozoa following plasma exposure (Fig. 2g–l).

**Table 5:** DNA methylation level (%) of chicken spermatozoa.

Exposure potential (kV)	*NRF2*	*KEAP1*	*PRDX4*	*ATP5A1*	*AMPKα2*	*mTOR*
0	32.89	34.46	36.10	26.73	25.34	26.56
11.7	23.16	41.96	26.36	20.96	31.21	20.16
27.6	48.42	21.09	50.26	38.27	13.97	37.05

Plasma exposure time was 20 s. The cytosine methylation sequencing results are shown in Fig. 2.

### Sperm mRNA and protein expression

**Figure 3:**
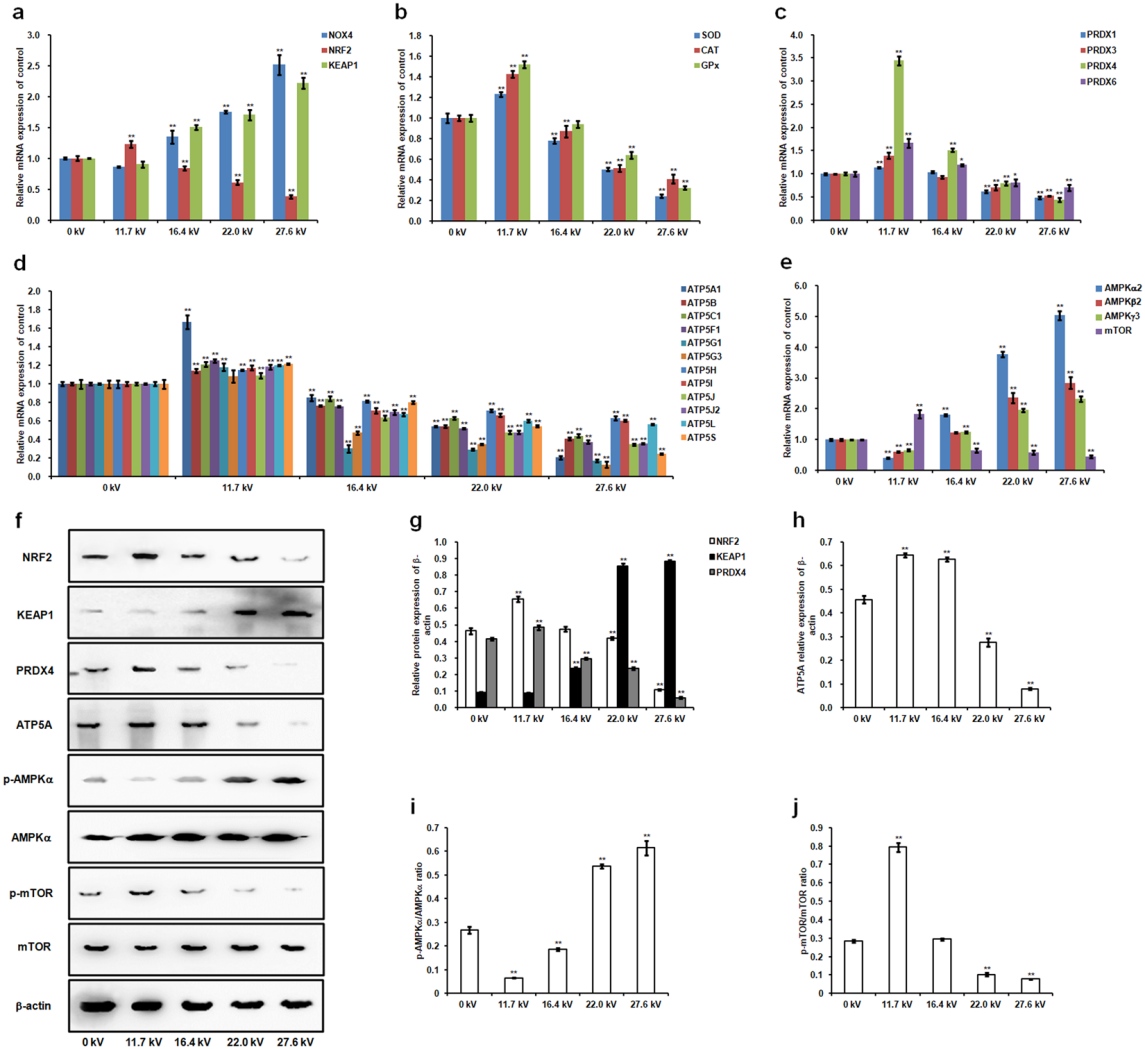
Effect of plasma on chicken sperm mRNA and protein expression. Semen of 60-week-old cocks was exposed to varying plasma potentials for 20 s. Relative mRNA levels of the following genes were measured: (**a**) *NOX4*, *NRF2*, and *KEAP1*; (**b**) *SOD*, *CAT*, and *GPx*; (**c**) *PRDX1*, *PRDX*3, *PRDX4*, and *PRDX6*; (**d**) *ATP5A1*, *ATP5B*, *ATP5C1*, *ATP5F1*, *ATP5G1*, *ATP5G3*, *ATP5H*, *ATP5I*, *ATP5J*, *ATP5J*2, *ATP5L*, and *ATP5S*; and (**e**) *AMPKα2*, *AMPKβ2*, *AMPKγ3*, and *mTOR*. (**f**) Western blot analysis of protein bands. Uncropped immunoblot scans are presented in Supplementary Figure S1. The grouping of gels/blots cropped from different gels. All blots were visualized with 5 min exposure time. Relative protein levels of (**g**) NRF2, KEAP1, PRDX4, (**h**) ATP5A, (**i**) p-AMPKα/AMPKα, and (**j**) p-mTOR/mTOR. Values are expressed as the mean ± standard error (n = 3) of three replicates; n represents an individual cock. ^*^p < 0.05 versus control; ^**^p < 0.01 versus control, according to one-way ANOVA and LSD test.

Plasma exposure exerts a dose-dependent effect on mRNA expression of antioxidant defense-related genes and energy metabolism-related genes and their protein levels in chicken spermatozoa (Fig. 3). At 20 s of exposure and 11.7 kV of plasma potential, we observe mRNA upregulation of nuclear factor erythroid 2-related factor 2 (*NRF*2), *SOD*, *CAT*, *GPx*, peroxiredoxins (*PRDXs*), *ATP* synthase subunits, and mammalian target of rapamycin (*mTOR*), but reduced mRNA expression of nicotinamide adenine dinucleotide phosphate oxidase 4 (*NOX4*), kelch-like ECH associated protein 1 (*KEAP1*), and AMP-activated protein kinase (*AMPK*) (Fig. 3a–e). This plasma treatment results in significant upregulation of NRF2, PRDX4, ATP5A, and mTOR phosphorylation, with increases of 0.41- (*p* < 0.001; Fig. 3f,g), 0.17- (*p* < 0.001; Fig. 3f,g), 0.41- (*p* < 0.001; Fig. 3f,h), and 0.79-fold (*p* < 0.001; Fig. 3f,j). On the other hand, AMPKα phosphorylation decreases 0.75-fold (*p* < 0.001; Fig. 3f,i), but no significant decrease is found on KEAP1 protein expression (Fig. 3f,g). However, further increases in plasma potential produce significant reversed effects on gene and protein expression.

## Discussion

Our results show that plasma exposure at 11.7 kV within 40 s results in an increase of sperm motility, without corresponding significant changes in sperm morphology, viability, integrity of acrosome and DNA, and total fertility. However, exposure time longer than 1 min or treatment with higher potentials reduces sperm parameters. In the previous study, we find that non-thermal DBD plasma affects chicken embryonic development in a dose-dependent manner during the early stage of incubation^11^. *In vitro* experiments using fibroblasts^24^, endothelial cells^3^, epithelial cells^5^, myoblast cells^25^, and tumor cells^26^ demonstrate the dose-dependent effects of plasma. Furthermore, longer durations of plasma exposure increase apoptotic cell number^27^, decrease cell viability^28^, and cause endothelial cell toxicity^3^. Based on these findings, we conclude that plasma affects chicken sperm motility in a time- and dose-dependent manner. We also show that optimal plasma conditions for increasing sperm motility are plasma potential of 11.7 kV and exposure time of 20 s.

Sperm quality is affected by ROS directly in a dose- and time-dependent manner^29^. Diffusion of plasma-generated ROS or stimulation of intracellular ROS-generating mechanisms as a result of non-thermal DBD plasma treatment^30^ has been suggested to regulate sperm quality and physiology^31^. ROS homeostasis between generation and scavenging activity is favorable to the sperm quality^16^. Low physiological concentrations of ROS *in vivo* are known to stimulate sperm capacitation and acrosome reaction^1,31^, whereas excessive ROS accumulation results in lipid peroxidation of sperm membrane, which in turn leads to impaired viability, motility and acrosome integrity, DNA damage, and infertility^16,32,33^. Our results show that plasma exposure at 11.7 kV for 20 s maintains relatively low ROS level and suppresses MDA activity, which are mediated by the upregulation of antioxidant enzyme activity of SOD, CAT, GPx, and PRDX, protecting against oxidative damage and increasing the viability of spermatozoa^1,26,34^. Furthermore, we find the optimal plasma treatment reduces mRNA expression of *NOX4*, which in turn inhibits ROS production^35–37^, as well increases NRF2 level to maintain ROS at a relative low level by regulating the antioxidant defense system^38–40^, further improves the chicken sperm motility. On the other hand, inappropriate plasma treatments degrade the sperm quality as a result of excessive ROS accumulation and impair the antioxidant defense system through disruption of the NRF2-antioxidant signaling pathway.

Sperm motility requires high ATP levels as the energy source^1^. Mitochondria have a crucial role in the energy metabolism and ATP generation^41^. Sperm motility is found to be directly and positively correlated with mitochondrial respiratory enzyme activity and ATP production in roosters^42^. Our results show plasma conditions can be optimized to enhance chicken sperm motility by increasing the activity of respiratory enzyme and ATP production through upregulation of the mRNA expression of *ATP* synthase subunits and ATP5A protein level in spermatozoa. Meanwhile, intracellular ROS generation is increased as byproducts in the mitochondrial respiratory chain^43^, but this do not induce an impairment of sperm quality because of the elevated scavenging activity of antioxidant enzyme which is mediated by an appropriate plasma exposure. However, inappropriate plasma treatment leads to mitochondrial enzymatic dysfunction^44,45^ as a result of disruption of ATP production in the mitochondria^46^, resulting in axonemal damage, decreased sperm viability and motility, and more sever morphological defects in the midpiece, with corresponding deleterious effects on sperm capacitation and acrosome reaction^32,47^. Therefore, appropriate plasma exposure conditions improve sperm energetic metabolism through mitochondrial ATP generation. In addition, our results reveal that the optimal plasma treatment decreases *AMPK* mRNA levels and AMPK phosphorylation, increases *mTOR* mRNA levels and mTOR phosphorylation in spermatozoa. These results can be explained by the fact that high ATP concentrations inactivate AMPK, which phosphorylates and activates the mTOR pathway^48^, further regulating intracellular energy metabolism^49^ and sperm motility and survival^50,51^. However, the activation of AMPK and decrease of mTOR expression result in low sperm viabilities in the inappropriate plasma-treated groups because of the cell apoptosis effect regulated by AMPK-mTOR pathway^52,53^.

In this study, we examine DNA methylation levels of antioxidant defense-related and energetic metabolism-related genes. The results show exposure to 11.7 kV of plasma for 20 s increases the demethylation levels of *NRF*2, *PRDX4*, *ATP5A1*, and *mTOR* but decreases demethylation levels of *KEAP1* and *AMPKα*2, resulting in the alteration of abovementioned gene expression in chicken spermatozoa because DNA demethylation has been associated with the gene transcriptional activation^54^, whereas hypermethylation is known to suppress gene expression^55,56^. Sperm DNA methylation levels are associated with sperm motility and DNA integrity, but not with sperm viability or morphology^18^. Sperm DNA hypermethylation results in impaired morphology^57^, low sperm motility^17,20^, infertility^58^, and even sperm apoptosis^59^. In the present study, optimal plasma-treated spermatozoa with DNA hypomethylation levels of *NRF*2, *PRDX4*, *ATP5A1*, and *mTOR* and hypermethylation levels of *KEAP1* and *AMPKα*2 exhibit an increase of sperm motility, but no changes in the sperm viability, acrosome and DNA integrity, morphology, and total fertility. On the other hand, high-intensity plasma treatment shows significantly reversed methylation patterns of the abovementioned genes and results in impaired sperm quality. Moreover, sperm DNA methylation influences DNA integrity and stable transmission of epigenetic information to the offspring^60^, which are crucial for embryonic development and post-natal growth^61^. These results demonstrate that plasma treatment of chicken spermatozoa may promote higher motility for next-generation poultry breeding.

In conclusion, exposure to 11.7 kV of plasma for 20 s enhances chicken sperm motility by regulating the demethylation levels of antioxidant defense-related and energetic metabolism-related genes in spermatozoa. Notably, optimal plasma treatment may potentially boost offspring production and thus warrant further investigation.

## Materials and Methods

### Plasma treatment

Fresh semen was collected from ten healthy cocks (Korean native chicken, approximately 60 weeks old; 10 cocks were used) that were raised in a chicken farm (Jeju National University, Jeju, Republic of Korea), following the method described by Blesbois, *et al*.^62^. Animal handling protocols were approved by the Institutional Committee for Ethics in Animal Experiments of Jeju National University and all experiments were performed in accordance with the institution guidelines. Individual ejaculates were then diluted at a 1:1 ratio with Beltsville Poultry Semen Extender^63^ in a sterile petri dish, and this dilution was performed within 10 min of collection. The semen sample was then placed horizontally on the glass dielectric barrier of the plasma reactor (Fig. 1f), allowing the semen to be directly exposed to the plasma propagating from the tip of the electrode needles at varying durations and potentials following our previously described method^10^. Semen without plasma treatment was used as the control group. Plasma-treated semen samples were kept at 37 °C in an incubator for a few minutes until further analysis.

### Sperm quality evaluation

Individual ejaculates were prepared at a 1:1 dilution in Beltsville Poultry Semen Extender^63^ and then a 1:30 dilution with 0.9% sodium chloride solution was performed prior to the sperm quality evaluation. Sperm count was measured using a hemocytometer according to World Health Organization criteria^64^. The evaluation of sperm motility was performed by two experienced individuals following World Health Organization criteria^64^. After liquefaction, 10 μl of diluted semen was pipetted onto a glass slide and covered with a cover slip. The analysis was performed at room temperature under a Leica DM 2500 microscope at a magnification of 400 ×. Sperm motility was calculated as the percentage of spermatozoa with linear and progressive motility^62^. Following liquefaction, 10 μl of diluted semen was spread onto a glass slide and allowed to air-dry at room temperature. Sperm vitality was analyzed by staining the smears with eosin Y. The evaluation was performed by counting the red-stained and unstained spermatozoa with brightfield optics at a magnification of 400 ×. Sperm vitality was expressed as the proportion (%) of unstained spermatozoa, and at least 100 spermatozoa were counted^64^. Semen smears were stained with Wright-Giemsa^65^, and sperm morphology was photographed using a Leica DM 2500 microscope equipped with a digital camera (DP71, Olympus) at a magnification of 1,000 ×. Acrosome integrity was calculated as the proportion (%) of acrosome covering 40% to 70% of the head area^64^. Semen smears were fixed in 96% ethanol-acetone (1:1) at 4 °C for 1 hour and air dried, then hydrolyzed in 0.1 N HCl at 4 °C for 5 min. Thereafter, the toluidine blue staining was performed as previously described by Kim, *et al*.^66^. Sperm nuclear DNA integrity was calculated as the percentage of spermatozoa with light blue-stained heads, and at least 200 spermatozoa were counted. Acrosome integrity and DNA integrity were analyzed under a Leica DM 2500 microscope using oil immersion with magnification of 1,000 ×.

Prior to artificial insemination (AI), healthy hens (Hyline brown chicken, raised at a chicken farm in Jeju National University, Jeju, Republic of Korea) that did not undergo AI nor naturally mated were randomly allocated into nine groups comprising ten hens per group. AI was performed twice a week with fresh non-plasma (control group) and plasma-treated semen at varying durations and potentials. To determine the total fertility for a five-day collection of fertilized eggs after AI, egg shells were wiped with 70% ethanol, incubated for 7 days at 37.5 °C under 45% to 65% relative humidity, and rotated 90 degrees every 2 h. Afterwards, breakout analysis of all incubated eggs was performed to check the number of embryos. Total fertility was calculated by dividing the number of embryos by the total number of incubated eggs in each group; values were expressed as percentages (%).

### ROS, antioxidant enzyme, and ATP analyses

Chicken semen was centrifuged at 600 × *g* for 10 min at 4 °C. Sperm pellets were washed with 0.9% sodium chloride solution, centrifuged again at 600 × g for 10 min at 4 °C, and suspended in 1 × phosphate buffered saline (PBS; 0.05 mol/l, pH 7.4) at a final concentration of 1 × 10^9^ spermatozoa/ml. ROS levels were determined using the OxiSelect *In Vitro* ROS/RNS Assay Kit (Cell Biolabs, Inc., San Diego, CA, USA). The assay employed a proprietary quenched fluorogenic probe 2′,7′-dichlorodihydrofluorescein DiOxyQ (DCFH-DiOxyQ), which is a specific ROS/RNS probe that is based on similar chemistry to 2′,7′-dichlorodihydrofluorescein diacetate. The DCFH-DiOxyQ probe is first primed with a quench removal reagent, and subsequently stabilized in the highly reactive DCFH form, which is rapidly oxidized to the highly fluorescent 2′,7′-dichlorodihydrofluorescein (DCF) by ROS/RNS. The amount of DCF in the sample is determined based on the relative fluorescence units obtained using a DCF standard curve and the fluorescence intensity of DCF is proportional to the total ROS/RNS level within the sample, using a GloMax Discover Multimode Detection System fluorescence plate reader (Promega, Madison, WI, USA) at 480 nm excitation/530 nm emission. ROS levels in the spermatozoa were expressed as nmole DCF/10^9^ spz.

Spermatozoa were assayed for MDA, SOD, CAT, GPx, and ATP levels using kits from Invitrogen (Thermo Fisher Scientific, Waltham, MA, USA) and Sigma-Aldrich (St. Louis, MO, USA) following the manufacturer’s instructions. The optical densities were determined using a GloMax Discover Multimode Detection System (Promega). The relative light unit value of ATP levels were measured using a luminometer (Sirius L Tube Luminometer, Titertek Berthold, Germany). SOD activity was expressed as U/10^9^ spz, where one unit was defined as the amount of enzyme that reduces 1.0 µmole of superoxide to molecular oxygen and hydrogen peroxide per min at 25 °C. CAT activity was expressed as U/10^9^ spz, wher U was defined as the amount of enzyme that decomposes 1.0 µmole of hydrogen peroxide to oxygen and water per min at 25 °C. GPx activity was expressed as mU/10^9^ spz, where mU was defined as the amount of enzyme that will cause the oxidation of 1.0 nmole of NADPH to NADP^+^ under the assay kit condition per min at 25 °C. MDA and ATP levels were expressed as nmole/10^9^ spz.

### Mitochondrial respiratory enzyme analysis

Mitochondria were isolated and purified from chicken spermatozoa (1 × 10^9^ spermatozoa/ml) using Qproteome Mitochondria Isolation Kit (QIAGEN, Valencia, CA, USA) according to the manufacturer’s instructions. The NADH levels and enzymatic activities of cytochrome c oxidase and ATP synthase in sperm mitochondria were measured using NAD+/NADH Quantitation Colorimetric Kit (BioVision, Milpitas, CA, USA), Cytochrome Oxidase Activity Colorimetric Assay Kit (BioVision), and ATP Synthase Activity Assay Kit (Novagen, Merck KGaA, Darmstadt, Germany) according to the manufacturer’s instructions, respectively. The optical densities of NADH, cytochrome c oxidase, and ATP synthase were measured using the GloMax Discover Multimode Detection System.

### RT-PCR analysis

Total RNA isolation and purification from spermatozoa were performed as previously described by Shafeeque, *et al*.^67^. RT-PCR analysis was performed using SuperScript III First-Strand Synthesis System for RT-PCR (Invitrogen), 2× Prime Taq Premix (GENETBIO, Yuseong-gu, Daejeon, South Korea), and EvaGreen Dye (Biotium, Hayward, CA, USA). First-strand cDNA synthesis was performed using 1 μl of total RNA (1 μg/μl) following the manufacturer’s protocol. RT-PCR was performed using a StepOne Real-time PCR system (Applied Biosystems, Thermo Fisher Scientific). The following conditions were used for RT-PCR: 40 cycles of initial denaturation at 95 °C for 10 min, followed by denaturation at 95 °C for 15 s and annealing for 1 min at the indicated temperatures shown in Supplementary Table S3. Melting curves were obtained at 95 °C for 15 s, 60 °C for 1 min, and increased to 95 °C for 15 s at increments of 0.3 °C. Primer sequences used for RT-PCR are shown in Supplementary Table S3.

The cycle threshold value for each sample was determined based on triplicate measurements. Equivalent dilutions were calculated according to the standard curve and then normalized against the housekeeping gene (*β-actin*). Reaction product sequences were confirmed by direct nucleotide sequencing using an ABI PRISM 7700 Sequence Detector (Applied Biosystems). Relative expression levels were calculated using the 2^−ΔΔCT^ method.

### Bisulfite conversion and bisulfite-sequencing PCR

Genomic DNA was extracted and purified from chicken spermatozoa using an AllPrep DNA/RNA Micro Kit (QIAGEN) following the manufacturer’s instructions as previously described by Kito, *et al*.^68^. Sodium bisulfite conversion was performed using 1 μl of genomic DNA (1 μg/μl), 19 μl of RNase-free water, 85 μl of Bisulfite mix, and 35 μl of DNA protect buffer with a Master Cycler Gradient (Eppendorf, Hamburg, Germany) following to the manufacturer’s instructions of the EpiTech Bisulfite Kit (QIAGEN). Bisulfite-sequencing PCR (BSP) amplification was performed using 1 μl of purified bisulfite-converted DNA (50 ng/μl), 10 μl of 2× Prime Taq Premix, 1 μl of upstream primer (5 pmol/μl), 1 μl of downstream primer (5 pmol/μl), and 7 μl of DEPC-treated water. Primers for BSP were designed using the MethPrimer (http://www.urogene.org/methprimer/) (see Supplementary Table S4). BSP amplification products were purified using the MiniBest Agarose Gel DNA Extraction Kit (TaKaRa Bio Inc., Kusatsu, Shiga, Japan) following the manufacturer’s instructions.

### Cloning and methylation sequencing

Purified BSP amplification products were ligated to the pGEM-T Easy Vector and transformed into competent JM109 cells using the pGEM-T Easy Vector system I (Promega). White colonies were selected from the ampicillin/X-Gal/IPTG plates, and colony PCR was performed using vector-directed primers to confirm the presence of inserts based on their expected fragment sizes. For plasmid isolation, ten positive colonies from each plate were inoculated into LB medium containing ampicillin. Plasmids containing the target DNA were extracted and purified using the Plasmid Midi Kit (QIAGEN) and then sequenced on an ABI Prism BioDye Terminator version 3.1 sequencing system. Cytosine methylation results were analyzed using the CyMATE online methylation sequence analysis software. Total methylation ratios were calculated by dividing the number of non-converted (methylated) cytosines by the total number of cytosines within the sequenced region; values were expressed as percentages (%). Average methylation levels were expressed as percentage (%) per site for each of the three types of cytosines (CG, CHG, and CHH), and were calculated by dividing the number of non-converted cytosines by the total number of cytosines of each type.

### Western blotting

Chicken sperm protein was extracted following the methods of Labas, *et al*.^69^. Protein concentration was determined using a bicinchoninic acid protein assay kit (Sigma-Aldrich) using bovine serum albumin (BSA) as standard. Proteins were separated by 12% sodium dodecyl sulfate-polyacrylamide gel electrophoresis and then transferred to polyvinylidene fluoride membranes via wet electrophoretic transfer (Bio-Rad, Hercules, California, USA). Membranes were blocked in PBS-0.08%Tween containing 5% dried skim milk or 3% BSA for 2 h at 25 °C, and subsequently incubated with primary antibodies at 4 °C overnight. The following antibodies were used: anti-NRF2 (mouse monoclonal; Santa Cruz Biotechnology; 1:200), anti-KEAP1 (mouse monoclonal; Santa Cruz Biotechnology; 1:200), anti-PRDX4 (mouse monoclonal; Santa Cruz Biotechnology; 1:200), anti- ATP5A (rabbit polyclonal; Abcam; 1:250), anti-phospho-AMPKα (Thr172, p-AMPKα; rabbit polyclonal; Cell Signaling Technology; 1:1,000), anti-AMPKα (rabbit polyclonal; Cell Signaling Technology; 1:1,000), anti-phospho-mTOR (Ser2448, p-mTOR; rabbit monoclonal; Cell Signaling Technology; 1:1,000), anti-mTOR (rabbit polyclonal; Cell Signaling Technology; 1:1,000), anti-beta actin (rabbit polyclonal; Bioss; 1:1,000). Secondary antibodies were goat anti-mouse (Santa Cruz Biotechnology; 1: 5,000) and goat anti-rabbit (Abcam; 1: 5,000) immunoglobulin G conjugated to horseradish peroxidase. Membranes were rinsed three times for 5 min. After incubation with the secondary antibody for 2 h at 25 °C, proteins were visualized via SuperSignal West Pico Plus Chemiluminescent Substrate (Thermo Fisher Scientific). Band intensities were quantified using ImageJ (Rasband WS, National Institutes of Health). Densitometric values of the NRF2, KEAP1, PRDX4, and ATP5A signals were normalized against the relevant β-actin signal. Densitometric values of the p-AMPKα, AMPKα, p-mTOR, and mTOR bands were normalized against the β-actin signal in the same sample before calculating the p-AMPKα/AMPKα and p-mTOR/mTOR ratios.

### Statistical analysis

All experiments were repeated three times, and data are presented as the mean ± standard error. Statistical analyses were performed using the Statistical Package for the Social Sciences (SPSS version 16.0; SPSS, Chicago, IL, USA). Statistically significant difference among treatment groups were determined via one-way ANOVA and Fisher’s least significant difference (LSD) test. *P*-values < 0.05 are considered significant.

### Data availability

The datasets generated during and analysed during the current study are available from the corresponding author on reasonable request.

## Electronic supplementary material

Supplementary Information
