# Supplementary material for: Non-thermal plasma treatment improves chicken sperm motility via the regulation of demethylation levels
Source: Sci Rep. 2018 May 15;8:7576. doi: 10.1038/s41598-018-26049-5 (PMC5953930; doi:10.1038/s41598-018-26049-5)
Supplement: Supplementary file 1 — Supplementary Information [file 41598_2018_26049_MOESM1_ESM.pdf]

Supplementary Information

Non-thermal plasma treatment improves chicken sperm motility via the regulation of demethylation levels

Jiao Jiao Zhang[1], Huynh Luong Do[1], Nisansala Chandimali[1], Sang Baek Lee[2], Young Sun Mok[2], Nameun Kim[1], Seong Bong Kim[3], Taeho Kwon[4,*], Dong Kee Jeong[1,4,*]

[1] Laboratory of Animal Genetic Engineering and Stem Cell Biology, Department of Advanced Convergence Technology and Science, Jeju National University, Jeju 63243, Republic of Korea

[2] Department of Chemical and Biological Engineering, Jeju National University, Jeju 63243, Republic of Korea

[3] Plasma Technology Research Center, National Fusion Research Institute, Gunsan-si, Jeollabuk-Do 54004, Republic of Korea

[4] Laboratory of Animal Genetic Engineering and Stem Cell Biology, Subtropical/Tropical Organism Gene Bank, Jeju National University, Jeju 63243, Republic of Korea

[*]Correspondence and requests for materials should be addressed to D.K.J. (E-mail: ngejeong@gmail.com) and T.K. (E-mail: taehokwonk@gmail.com)

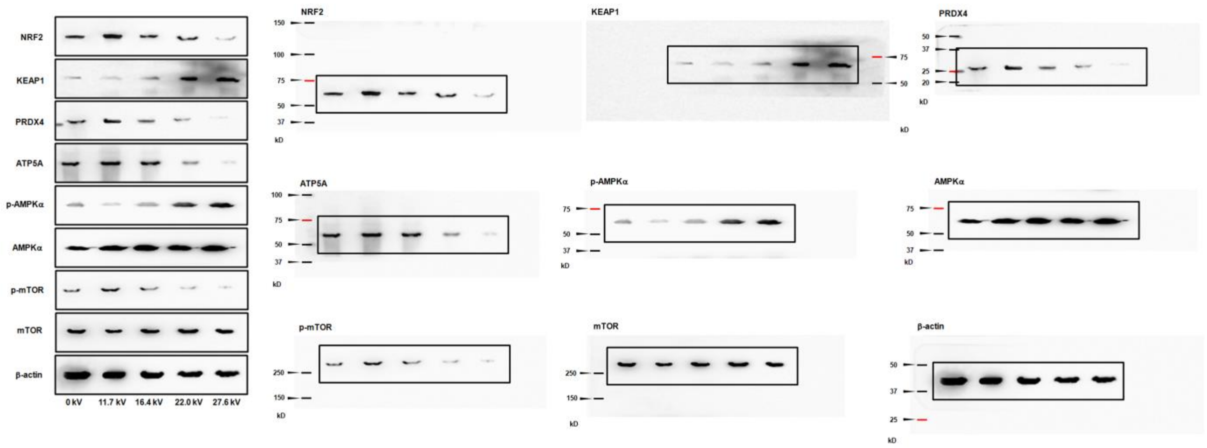

Supplementary Figure S1. Western blot analysis of the protein levels of NRF2, KEAP1, PRDX4, ATP5A, p-AMPKα, AMPKα, p-mTOR, and mTOR in chicken spermatozoa exposed to different potentials for 20 s.

Uncropped immunoblot scans for Figure 3f. The grouping of gels/blots cropped from different gels. All blots were visualized with 5 min exposure time.

Supplementary Table S1. Effect of plasma exposure for different durations on ROS and antioxidant enzyme in chicken spermatozoa

Exposure duration (s)	ROS (nmole DCF/10^9spz)	MDA (nmole/10^9spz)	SOD (U/10^9spz)	CAT (U/10^9spz)	GPx (mU/10^9spz)
0	6.47±0.18	3.04±0.18	42.53±0.91	2.16±0.11	4.34±0.10
10	6.07±0.04*	2.66±0.17	67.48±2.61**	2.85±0.17**	5.86±0.16**
20	5.79±0.18*	2.04±0.17**	72.64±3.17**	3.45±0.23**	6.15±0.31**
40	6.20±0.10	2.76±0.18	58.42±2.35**	2.64±0.14**	5.44±0.25**
60	7.61±0.19**	3.35±0.27	40.37±2.29	1.85±0.07	4.13±0.10
80	8.65±0.08**	3.85±0.18**	33.58±0.80**	1.65±0.10**	3.54±0.17**

Plasma exposure intensity was 11.7 kV. Values are expressed as the mean ± standard error (n=10) of three replicates; n represents an individual cock. Within a column: *, $p < 0.05$ versus control; **, $p < 0.01$ versus control, according to one-way ANOVA and LSD test.

Supplementary Table S2. Effect of plasma exposure for different durations on ATP and activity of mitochondria respiratory enzyme in chicken spermatozoa

Exposure duration (s)	ATP (nmole/10^9spz)	NADH (U/10^9spz)	Cytochrome c oxidase (mU/10^9spz)	ATP synthase (mU/10^9spz)
0	3.62±0.15	0.57±0.02	34.55±0.93	21.53±0.93
10	4.85±0.10**	0.68±0.04**	46.74±1.66**	27.47±1.80**
20	5.84±0.40**	0.79±0.02**	56.40±3.16**	31.30±2.38**
40	4.64±0.16**	0.71±0.02**	49.46±2.44**	28.46±1.75**
60	3.26±0.10	0.50±0.05*	30.40±1.52	19.32±0.80
80	2.74±0.10**	0.42±0.01**	24.48±1.69**	16.37±1.80**

Plasma exposure intensity was 11.7 kV. Values are expressed as the mean ± standard error (n=10) of three replicates; n represents an individual cock. Within a column: *, $p < 0.05$ versus control; **, $p < 0.01$ versus control, according to one-way ANOVA and LSD test.

Supplementary Table S3. Primer sequences for the RT-PCR

Gene	Sequence number	Sequence position	Product length (bp)	Annealing Temperature (°C)	Sequence (5'to3')
β-actin	NM_205518.1	625-818	194	57	F: GTGCGTGACATCAAGGAGAAGC<br>R: CCACAGGACTCCATACCCAAGA
NOX4	NM_001101829.1	28-157	130	57	F: CGAGGATCTCAGAAGGTTGC<br>R: GAGCATTCACCAGATGAGCA
NRF2	NM_205117.1	484-619	136	57	F: AAAACGCTGAACCACCAATC<br>R: GCTGGAGAAGCCTCATTGTC
KEAP1	KU321503.1	1227-1485	259	57	F: GTATCACAGCAGCGTGGAGA<br>R: GGCGTAGATGCAGTTGTTGA
SOD	NM_205064.1	106-278	173	55	F: ATTACCGGCTTGTCTGATGG<br>R: CCTCCCTTTGCAGTCACATT
CAT	NM_001031215.2	1067-1276	210	55	F: CTCATTCCAGTGGGCAAGAT<br>R: GTAGGGGCAATTCACAGGAA
GPx	NM_001277853.2	353-474	122	55	F: ATGTTCGAGAAGTGCGAGGT<br>R: ATGATGTACTGCGGGTTGGT
PRDX1	NM_001271932.1	358-545	188	56	F: ACAAGGTGGTTTGGGCACTA<br>R: TCTCATCAACAGAACGGCCA
PRDX3	XM_426543.5	414-551	138	56	F: TTTCACCTTTGTGTGCCCCA<br>R: TTGCGCGGGGTATTTATCCA
PRDX4	XM_001233999.3	595-733	139	56	F: TGCACTTAGGGGCCTTTTCA<br>R: TTCTCCATGCTTGTCCGTGT
PRDX6	NM_001039329.2	189-340	152	58	F: TGAGTTCAGCAAACGCAACG<br>R: GCTCTCGGTCCTTATCAGCG
ATP5A1	NM_204286.1	1207-1364	158	57	F: GGTATCCGTCCAGCCATCAA<br>R: GCATCCAAATCAGACCCAAACT
ATP5B	NM_001031391.2	482-637	156	57	F: GCCCCATCACAACGAAACAG<br>R: CGCCTCCAAACAAACCAATC
ATP5C1	NM_001278096.1	272-411	140	57	F: ATTAAGGCACCCGAGGACAA<br>R: ACTTCCTTCCCTGCATTGGA
ATP5F1	XM_417993.4	437-644	208	57	F: CATTGGAGACTGCCATTGAGG<br>R: TGATCTTGCTCTTTCTGACGCTT
ATP5G1	XM_001233602.3	287-536	250	57	F: CAGGAGCAGGTATTGGGACA<br>R: TTGTCAGTCTGGAACGCTCT
ATP5G3	NM_001277855.1	141-288	148	57	F: CCAAAACGCTGTCTCCCAAC<br>R: ACCGAAGACCGTTCCAATACC
ATP5H	XM_001232598.3	332-551	220	57	F: CTGAAGGTCCCTGAACCAGT<br>R: ACTTCTCCCTGTCCAGTCTG
ATP5I	NM_001097534.2	74-240	167	57	F: TCTCGCCCCTCATCAAGTTC<br>R: TGCCAGTTCCTTTGCAATCC

ATP5J	XM_004938370.1	58-197	140	58	F: CACTTGCGGAGAAACATCGGT<br>R: CCTACATCAACAGGTCCTCCAGC
ATP5J2	NM_001257200.1	170-263	94	57	F: GCCTCGGTGGTATCAGTATGGT<br>R: TACTTCCTGCGGCGGTCAT
ATP5L	XM_015298211	250-377	128	57	F: CCATGGTCAGGAGCTTTCAG<br>R: GCCTCGTTTGCCTATGATCTC
ATP5S	NM_001277562.1	46-279	234	57	F: TCCCCTTCCCCTTTCTTTCC<br>R: CATAGCCTTGATAGCGCACC
AMPKα2	NM_001039605.1	726-943	218	57	F: GGAGGCGTGTTTTACATCCC<br>R: AACTTCTCACAGACCTCCCG
AMPKβ2	NM_001044662.1	435-661	227	57	F: CCAGTGTTTTCAGCTCCCAC<br>R: GAGGTCCAGGATAGCGACAA
AMPKγ3	NM_001031258.2	183-320	138	57	F: GCTGGAACCCGACAACAATT<br>R: GCCTTCTTGATCTCCAGGGT
mTOR	XM_417614.4	119-309	191	57	F: TGAAGGGGTCAAGGCAATCC<br>R: GGCGAGCAGTGGTTGTGGAT

Supplementary Table S4. Primer sequences for bisulfite-sequencing PCR

Gene	Sequence position	Product length (bp)	Annealing Temperature (°C)	Sequence (5'to3')	Expected No. of CpGs
NRF2	407-562	156	55	F: TTTGTTGGTTTAGTTGGTGTTAGAT<br>R: CAATCATCAAAAAACAAAACCTCTT	4
KEAP1	583-849	267	55	F: AATTTGTTTTATTGTTAGTTGGTTA<br>R: CAAAATAAAATCCTAAAAAATCTAC	30
PRDX4	70-343	274	56	F: GTTGTTGTTGGTTTTGTTGTTGTA<br>R: AAAAACAAAATATTTTCCTTCATAATCTAT	24
ATP5A1	1082-1292	211	55	F: GAGGTTTTTTGATTGTTTTGTTTGT<br>R: CCTACCACCTATTTCATTACCCTAAT	4
AMPKα2	233-463	231	52	F: AGAAATTTAAAATTTGAAATTTTTT<br>R: AAATCCCTATAAACAACCATATATC	10
mTOR	1690-1890	201	52	F: ATATTTAGGATGGGTTGTTGAAAAT<br>R: AAATCAAAAAATACCCTTCAAACTC	4
